# Temporal and spatial instability in neutral and adaptive (MHC) genetic variation in marginal salmon populations

**DOI:** 10.1038/srep42416

**Published:** 2017-02-10

**Authors:** Kate Ciborowski, William C Jordan, Carlos Garcia de Leaniz, Sofia Consuegra

**Affiliations:** 1 Institute of Zoology, Zoological Society of London, Regent’s Park, London, NW1 4RY, UK; 2 Department of Biosciences, College of Science, Swansea University, Swansea SA2 8PP, UK.

## Abstract

The role of marginal populations for the long-term maintenance of species’ genetic diversity and evolutionary potential is particularly timely in view of the range shifts caused by climate change. The Centre-Periphery hypothesis predicts that marginal populations should bear reduced genetic diversity and have low evolutionary potential. We analysed temporal stability at neutral microsatellite and adaptive MHC genetic variation over five decades in four marginal Atlantic salmon populations located at the southern limit of the species’ distribution with a complicated demographic history, which includes stocking with foreign and native salmon for at least 2 decades. We found a temporal increase in neutral genetic variation, as well as temporal instability in population structuring, highlighting the importance of temporal analyses in studies that examine the genetic diversity of peripheral populations at the margins of the species’ range, particularly in face of climate change.

The importance of marginal populations for the long-term maintenance of species’ genetic diversity and evolutionary potential has long been discussed^1^,^2^ but it is now particularly timely in view of range shifts caused by climate change^3^,^4^. According to the Centre-Periphery hypothesis, marginal populations inhabit unstable and poorly connected habitats, and may be expected to harbour less genetic variation and have lower evolutionary potential than those at the centre of the distribution^5^,^6^. However, although genetic diversity within populations seems to decline on average from the centre of the distribution to the periphery^6^, there is no conclusive evidence that geographically, historically or climatically marginal populations display lower average fitness^7^. Understanding the dynamics of populations at the species’ range limits, including patterns of extinction and recolonization and their ability to adapt to environmental variation, is key to predict their responses to climate change^8^. Critically, studies looking at genetic diversity in central versus marginal populations have largely focused on contemporary patterns of genetic diversity, using primarily neutral markers^6^, which may not fully reflect the adaptive potential of populations^9^.

The genes of the major histocompatibility complex (MHC), which are some of the most studied in relation to adaptive genetic variation^10^,^11^, are useful markers to use in combination with neutral markers to reconstruct not only the genetic diversity but also the adaptive potential of marginal populations. MHC genes are central to immunity as they encode for proteins that present pathogen-derived antigens to T-cells, initiating the adaptive immune response^12^. MHC genes are amongst the most polymorphic genes in vertebrates and also some of the best studied^13^ and the variation in the MHC residues that bind antigens from pathogens is thought to be maintained by balancing selection driven by pathogens^14^,^15^ and also influenced by mate choice^16^,^17^. Evidence of selection on the MHC genes has been identified in many species as heterozygote advantage^18^,^19^, association of individual MHC alleles and/or genotypes with susceptibility to specific pathogens^20^,^21^, rare-allele advantage^22^ and changes in allele frequencies under experimental infections^23^. Comparisons between MHC loci, or markers linked to them, and neutral markers can be used to infer differences in the relative levels of neutral and adaptive variation within and among populations, which can be variable not only across closely related species^24^,^25^ but also within species depending on the spatial scale of analysis^26^.

Salmonids are good study models of marginal populations because most of their current species’ range was recolonised from a few refugia after the last glaciation^27^,^28^,^29^, which permits geographical comparisons among recently diverged populations. Most salmonid species have declining populations in at least some parts of their range^30^,^31^,^32^,^33^ and within the salmonids, the Atlantic salmon (*Salmo salar*) is probably the species which has suffered the most dramatic decline^34^. The main causes for these declines are over-exploitation, habitat fragmentation, interactions with farm escapes^35^, and likely climate change^36^. In addition, as a result of their anadromous life cycle and homing behaviour, Atlantic salmon populations tend to be locally adapted^37^. Atlantic salmon is also particularly suited for MHC studies as it represents the minimal-essential-MHC in fish, with only two unlinked MHC class I and class II genes expressed^38^,^39^ and evidence of balancing selection acting on potential peptide binding residues (PBRs) in class I^39^ and in class II loci^26^,^38^. Previous studies had shown evidence of salmon MHC-disassortative mating^40^, as well as an association between specific MHC alleles and pathogen resistance^41^,^42^,^43^ and there are microsatellite markers linked to both class I and class II loci that can be used as good proxies for functional variation at these genes^44^,^45^,^46^.

In Europe, the current distribution of the Atlantic salmon seems to reflect a pattern of postglacial recolonisation from multiple refugia^28^,^47^. Iberian salmon populations inhabit the southern limit of the species’ range and the Iberian glacial refugium seems to have been one of the important refugia from which the northern Atlantic salmon range was recolonised^28^. Some of these marginal populations have suffered severe declines over the last 100 years, particularly during the last decades^48^,^49^, but seem to maintain their genetic distinctiveness^28^ and a higher genetic diversity than expected from their population size^50^.

Here, we examined temporal and spatial patterns of neutral and adaptive genetic diversity measured by microsatellites and MHC-linked markers in four marginal salmon populations in the Iberian refugium with a complicated demographic history, to test the general hypothesis that marginal populations have temporarily unstable genetic diversity and low adaptive potential. To this end, we compared spatial and temporal patterns of variability at neutral markers and markers linked to genes under selection (MHC class I and class II).

## Results

### Microsatellite variability and population structuring

**Figure 1:**
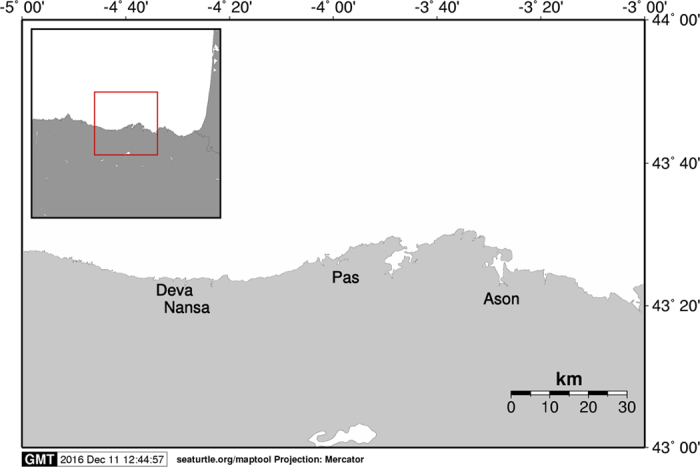
Location of the study rivers in Northern Spain. The distance between rivers varies from 3 to 50 Km. Map created using Maptool (http://www.seaturtle.org/maptool).

**Figure 2:**
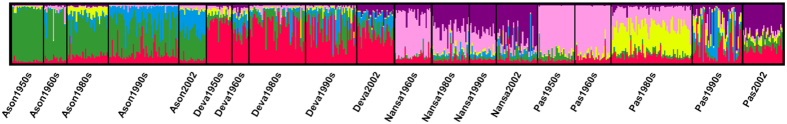
STRUCTURE clustering of Atlantic salmon adult samples (N = 598) from four marginal populations in Northern Spain over four decades. Each individual is represented by a vertical bar of a colour that represents its estimated membership to one of 6 genetic clusters. Labels below the plot indicate river and decade.

**Figure 3:**
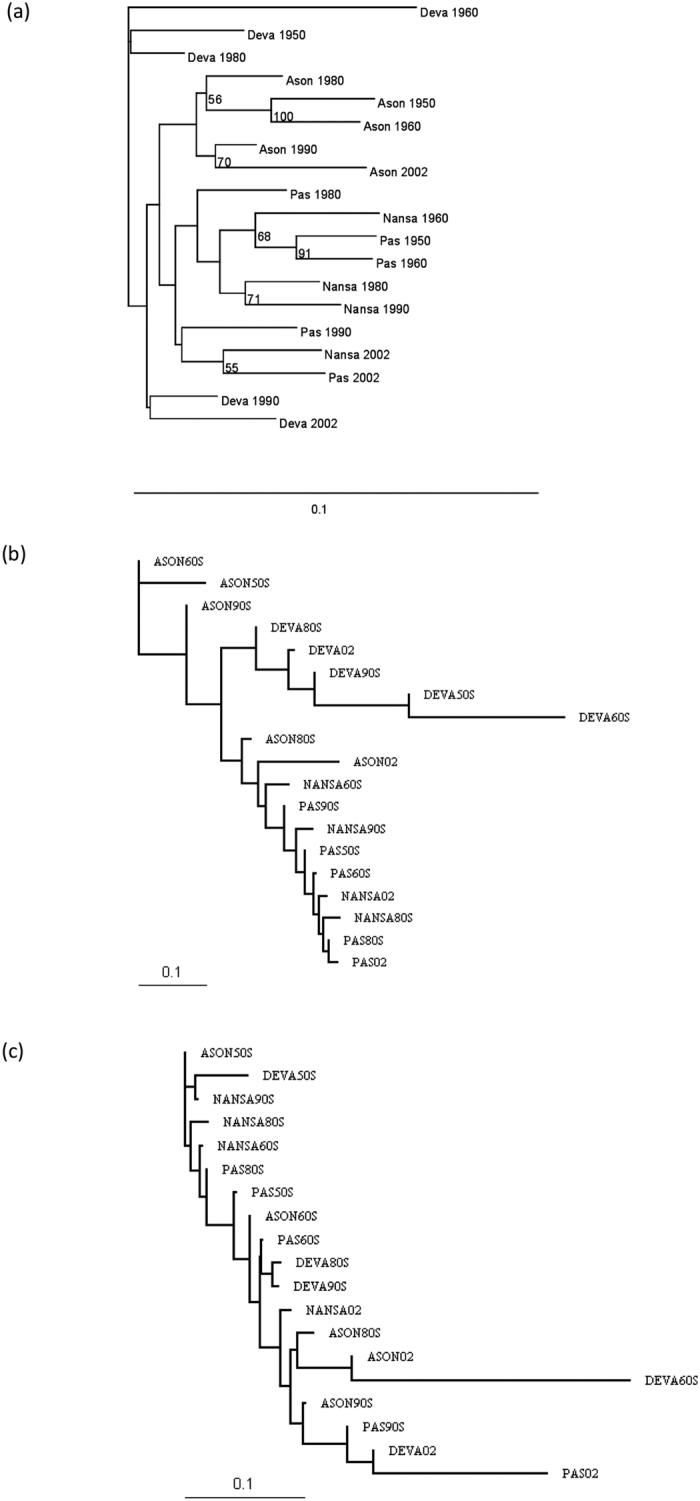
Neighbour-Joining (NJ) phylogenetic tree depicting the relationship between temporal samples from four different peripheral salmon populations based on (**a**) microsatellites, (**b**) *Sasa-UBA-3’UTR* MHC lined marker and (**c**) *Sasa-DAA-3*′*UTR* MHC lined marker. Numbers in the microsatellites tree indicate clusters supported by >50% of 5000 bootstrap iterations.

Individuals captured by anglers in the rivers Asón, Deva, Nansa and Pas (Northern Spain; Fig. 1) between 1948 and 2002 were genotyped at 13 putatively neutral microsatellite DNA markers. Deviations from HWE were only observed for loci CTAX (13 samples) and Sssp2210 (8 samples) following strict Bonferroni correction for multiple tests (Table 1). The overall results of the analyses did not change by excluding these two microsatellites (data not shown) and we opted for including them. A total of 196 alleles were observed across neutral microsatellites for the whole sample. BOTTLENECK results indicated that allelic frequency distributions did not depart from the expected L-shaped distribution. The number of alleles ranged from five at locus SsaD486 to 26 at locus SsaD144b. Significant correlations in allele frequencies between adjacent temporal samples for all comparisons could suggest stability in allele frequencies, but none of the correlations was significant after applying strict Bonferroni correction for multiple tests (Supplementary table S1). Heterozygosity (Ho) increased significantly over time in the rivers Pas (Mann-Kendall trend test s = 8 P = 0.041), Nansa (s = 6 P = 0.042) and Pas (s = 10, P = 0.008) whilst no significant temporal change in Ho was observed in the Deva (s = 6 P = 0.117). Equally, allelic richness increased temporally in the rivers Asón (s = 8 P = 0.042), Nansa (s = 6 P = 0.042) and Pas (s = 8 P = 0.042), but not in the river Deva (s = 6 P = 0.117). Following population analysis by STRUCTURE, the estimated optimal number of genetic groups was K = 6 (Fig. 2). The results indicated that the rivers Pas and Nansa, and to a lesser extent the river Ason, suffered a drastic change in population structure post-80 s, such that the genetic composition of these rivers in 2002 is rather different than that observed in 1950 and 1960. Similar results were obtained when the rivers were analysed individually (Supplementary figure S1). Genetic distance (D_A_) between temporal samples of the same rivers (0.057 to 0.177) were of a similar order to genetic distances between river samples (0.060 to 0.215). The NJ-phenogram, although with low statistical support, suggested that samples from the rivers Ason and Deva tended to group by river and not by decade, whereas samples from the rivers Nansa and Pas were intermingled, with a tendency to associate by decade instead of river (Fig. 3). AMOVA results also indicated significant temporal heterogeneity within the Asón and Pas samples (Table 2). Among river genetic variation was significant for each temporal sample, excluding the 1990 s samples, though there seemed to be a decrease in magnitude of F_ST_ over time. No deviation from neutrality was identified by the Ewens-Watterson test. The selection analysis implemented in LOSITAN identified outliers only in the samples from 1960 and 1980, in particular Ssa197 (P = 0.997), SsaD485 (P = 0.998), Ssa1438 (P = 0.984) in 1960, all under positive selection and Ssa197 (P = 0.997), SsaD485 (P = 0.997) in 1980, also under positive selection.

### MHC-linked variability and population structuring

Considering all historical samples, the rate of false alleles was low for both *Sasa-DAA-3*′*UTR* (0.28) and *Sasa-UBA-3*′*UTR* (0.30). Allelic drop out for *Sasa-DAA-3*′*UTR* was higher than reported for the neutral microsatellites (9.63 compared to the neutral mean of 3.89), whereas the ADO rate for *Sasa-UBA-3*′*UTR* was more similar (2.96). A total of 22 different alleles were found for the *Sasa-UBA-3*′*UTR* locus, and 15 for the *Sasa-DAA-3*′*UTR* locus. The large range of *Sasa-DAA-3*′*UTR* allele sizes (207 bp-367 bp) may have contributed to the greater rate of ADO, particularly in historical samples^46^. Nine samples significantly deviated from Hardy-Weinberg equilibrium after Bonferroni corrections (Table 1), most of them corresponding to the oldest samples, and did not appear to have any particular bias for either the *Sasa-UBA-3*′*UTR* locus or the *Sasa-DAA-3*′*UTR* locus. Analysis of linkage disequilibrium of class I and class II linked markers for all samples revealed no linkage disequilibrium of these loci (global P-value = 0.531). Observed heterozygosities ranged from 0.17–1.00 across all samples (Table 1). Time series analyses indicated an increase in genetic diversity in the river Pas in the *Sasa-DAA-3*′*UTR* locus (Ho and Ar: s = 8 P = 0.0042) and a decrease in *Sasa-UBA-3*′*UTR* in the rivers Nansa (Ho and Ar: S = −4 P = 0) and Pas (Ho: s = −1 P = 0.009; Ar: s = −2 P = 0.006). According to LOSITAN, *Sasa-UBA-3*′*UTR* locus was under positive selection in 1960 (P = 0.995) and 1980 (P = 0.995). In contrast to neutral microsatellites, significant correlations in allele frequencies were observed between adjacent temporal samples for all comparisons, except for those involving the Deva 1960s sample (Supplementary Table S1). This indicates stability of allele frequencies over time for both *Sasa-UBA-3*′*UTR* and *Sasa-DAA-3*′*UTR* loci. Allele distributions mostly overlapped among the four populations, with some very low frequency alleles being only represented in one or two of the rivers (Supplementary Figure S2). AMOVA analysis indicated significant genetic structuring at the class I locus at all temporal periods tested, whereas for the class II locus, the 1980s and 1990s were significantly differentiated as well as samples from 2002 and the 1960s (Table 3). The phylogenetic tree for the class I-linked marker indicated a relationship among rivers very similar to that for the neutral microsatellites, clustering the samples from rivers Asón and Deva according to river of origin, whereas the samples from rivers Nansa and Pas were largely intermingled within a cluster. In contrast, the class II marker, showed no structuring of samples based on river of origin in any of the rivers.

## Discussion

Peripheral (marginal) populations tend to be genetically and morphologically distinct as a consequence of their isolation and typically smaller size, and are considered particularly valuable because they can help preserve the evolutionary potential of the species^2^. Atlantic salmon populations in northern Spain represent peripheral populations at the southern limit of the species’ range; these have been in decline since the 1960’s and are now classified as endangered^51^. However, despite inhabiting the margins of the species’ range and having small effective population sizes, these populations display levels of genetic diversity comparable to those reported for larger populations at the center of the distribution^50^. Northern Iberian rivers are thought to have been a refugium for Atlantic salmon during the last glacial maximum, and it is possible that this is the reason why these populations appear to harbour higher than expected ancestral mitochondrial DNA variation compared to more northerly European populations^28^. Additionally, stocking from different sources carried out in the 80s and in the 90s could have also contributed to the temporal differentiation of these populations^31^. Between 1972 (when stocking records start) and the 90s, these rivers (initially the Rivers Ason and Pas and then extending to the River Nansa and to lesser extent the River Deva) were stocked with high densities of eyed ova (200,000-300,000 annually) and fry (90,000-120,000 annually) mainly from Scotland and Iceland. Stoking from the 90s was carried out from native sources, and primarily from the river of origin during the last years^48^,^51^. Our results from neutral microsatellites indicate that there has been a temporal increase in genetic diversity (heterozygosity and allelic richness) in three of the four rivers over a 50 year period, but also some temporal maintenance of genetic identity in the river Deva. In contrast, increases in neutral genetic diversity in the rivers Ason, Nansa and Pas, coupled with the strong changes in their genetic background from the 80s and a temporal decrease in genetic structuring suggest that their genetic composition could have been affected by foreign stocking, as previously indicated using mtDNA^31^. We found no conclusive evidence of selection in the MHC-linked markers, apart from *Sasa-UBA-3*′*UTR*, that together with three neutral microsatellites deviated from neutrality in samples from the 60s and 80s, suggesting the parallelism between the class I marker and the rest of the microsatellites. Results from neutral markers largely mirrored those of the class I MHC-linked marker (*Sasa-UBA-3*′*UTR*) but not those of *Sasa-DAA-3*′*UTR* (class II). This is perhaps not surprising given the differences in response to selection previously observed between both markers^46^. In this case, *Sasa-DAA-3*′*UTR* did not indicate any clustering of samples, by river or decade. Such a pattern of variation could reflect adaptation to local conditions in these marginal populations, an scenario that might be expected given the homing behavior of Atlantic salmon and their tendency to form locally adapted populations^37^, but also genetic drift due to low effective population size. Recently introduced salmonid populations in Chile suggested that MHC class II functional diversity of invasive populations has decreased over time, in contrast to diversity at neutral markers which has remained very high^52^,^53^ as a consequence of admixture^54^. Therefore, it is possible that, even if some neutral diversity has remained high in some Iberian salmon rivers, perhaps as consequence of foreign stocking, diversity at non-neutral markers may have been eroded over time due to geographical differences in selection^55^ and to adaptation to local conditions (e.g. parasites)^56^,^57^. Our current results, in combination with previous studies on the same populations, indicate, despite their low effective population size^50^, these salmon harbour high neutral genetic diversity, atypical in marginal populations, highlighting the importance of the demographic history for the maintenance of the genetic diversity. This is particularly relevant in view of the predictions of the consequences of climate change for salmonids, i.e. movement of the thermal niche of salmon towards north as well as decreased production and population extinction in the southern range of species^58^. Our study highlights the importance of a adopting not only a spatial but also a temporal approach, considering both neutral as well as adaptive markers, in studies that examine changes in genetic diversity of peripheral populations at the margins of the species’ range.

## Methods

### Origin of the samples and DNA extraction

Adipose fins from dead adult Atlantic salmon, captured by anglers in the rivers Asón, Deva, Nansa and Pas (Northern Spain; Fig. 1) were collected in 2002 and stored in 95% ethanol at 4 °C prior to genetic analysis. Dried scales from the same rivers collected from adult fish caught by anglers since 1948 were also included in the analyses. Due to their limited availability, historical scales were pooled across four decades following Ciborowski *et al*.^31^: 1948–1957, 1960–1963, 1980–1989, 1990–1996. No scale samples were available for any river from the 1970s, or for the river Nansa pre-1960. Therefore, 19 groups of samples, stratified by decade and river were generated for analysis (Table 1).

Total DNA was extracted using the Promega™ Wizard SV 96 Genomic DNA Purification System. Manufacturer’s protocols were adhered to for modern adipose fin samples, but for historical scales we increased the incubation time during the elution steps to five minutes and decreased the elution volume to 80–100 μl. Between one and three historical scale extractions were carried out in a dedicated ancient DNA laboratory, physically separated from PCR procedures. A blank control was extracted concurrently and subsequently amplified in PCR reactions. All eluted DNA was stored at −20 °C.

### PCR amplification and microsatellite genotyping

All individuals were genotyped at 13 putatively neutral microsatellite DNA loci (Ssa85, Ssa171, Ssa197, Ssa202^59^; SSsp1605, SSsp2210^60^; SsaA124, SsaD144, SsaD486^61^; SsoSL438^62^; CTAX, EST47, HSP^63^; Table 1) and two MHC linked markers, *Sasa-UBA-3*′*UTR* and *Sasa-DAA-3*′*UTR*^64^, in three multiplex reactions. Each individual was repeatedly genotyped at all loci, and 10% of the samples were genotyped in triplicate. Reactions were carried out according to the QIAGEN Multiplex PCR Kit reaction protocol in 8 ul volume. Each reaction included 4 ul of QIAGEN Multiplex PCR Kit reaction mixture, 2 mM of each primer and 2 ul of the extracted DNA solution. For the scale samples, 0.2 uM BSA was added to each reaction. The thermocycler profile consisted of 95 °C for 15 min, either 30 or 35 (tissue or scale DNA extraction, respectively) cycles of 94 °C 30 sec, 58 °C 90 sec, 72 °C for 30 sec and a final hold of 60 °C for 30 min. PCR products were run on a 3100 ABI Prism capillary sequencer using the Genescan-500 LIZ size standard. Alleles were scored using Genemapper V3.5 software (Applied Biosystems) and genotypes were manually checked.

### Data analysis

Individuals with fewer than eight successfully genotyped loci were discarded from analysis (final sample sizes in Table 1). All loci were tested for conformity with Hardy-Weinberg equilibrium using the randomization test implemented in GENEPOP^65^ and were also tested for neutrality using the Ewens-Watterson test^66^ in ARLEQUIN v3^67^. Rates of allelic dropout (ADO) and false alleles (FA) were estimated according to Broquet and Petit (2004)^68^. Allelic richness (Ar) was calculated using FSTAT^69^. Statistical significance of temporal trends was tested using the Mann-Kendall trend test^70^ implemented in PAST^71^. Pairwise differences in allelic frequencies between decades were estimated for each river using GENEPOP. F_ST_ values of genetic differentiation were estimated using GENETIX v.4.04^72^ and significances were determined with 1000 permutations To correct for simultaneous tests, strict Bonferroni corrections were applied^73^.

STRUCTURE v2.3.3^74^ was used to test how many genetic populations were represented by all individuals caught in each of the four rivers. We followed the methodology outlined in^74^. First, we constructed phylograms for all individuals from each river based on individual distance matrices calculated with the program POPULATIONS^75^ using an allele sharing distance (ASD) method^76^ to visualise whether there was any clustering of individuals into discrete population units. Following this, all individuals from each river were modelled in STRUCTURE. The program was run applying the admixture model, as this model was likely to be closer to the true nature of the history of these populations compared with a non-admixture model. The parameters of the simulations were burn-in length of 50,000 iterations; 100,000 MCMC repetitions; testing for K (the number of populations) between 2 and 8 over 10 repeated simulations. We estimated the correct value of K using the Evanno method^77^ as implemented in STRUCTURE HARVESTER (http://users.soe.ucsc.edu/~dearl/software/struct_harvest/). We then used CLUMPP^78^ and DISTRUCT^79^ to summarise and represent the results. Spatial and temporal structuring was also analysed using AMOVA as implemented in ARLEQUIN and POPULATIONS was used to generate a consensus unrooted neighbour-joining tree (10,000 bootstrapped replicated) of the samples based Nei’s D_A_ distance^80^, which was visualized using TREEVIEW^81^.

*Sasa-UBA-3*′*UTR* and *Sasa-DAA-3*′*UTR* data were analysed separately (as in^64^). GENEPOP on the web^82^ was used to estimate observed and expected heterozygosities (H_o_ and H_e_) for each locus in each sample. Allelic richness was calculated at each locus for each sample using FSTAT version 2.9.3^83^. Statistical significance of the temporal trends of genetic diversity (heterozygosity and allelic richness) was tested using the Mann-Kendall trend test^70^ implemented in PAST^71^.

All markers, neutral and MHC-linked microsatellites, were tested for neutrality using Lositan^84^,^85^, under 50,000 simulations, estimated neutral mean F_ST_, infinite alleles mutation model, 99% confidence interval and false discovery rate of 0.1%. All populations were tested for recent bottlenecks using BOTTLENECK v.1.2.02^86^.

## Additional Information

**How to cite this article**: Ciborowski, K. *et al*. Temporal and spatial instability in neutral and adaptive (MHC) genetic variation in marginal salmon populations. *Sci. Rep.* **7**, 42416; doi: 10.1038/srep42416 (2017).

**Publisher's note:** Springer Nature remains neutral with regard to jurisdictional claims in published maps and institutional affiliations.

## Supplementary Material

Supplementary Information

## Figures and Tables

**Table 1:** Temporal and spatial measures of genetic diversity for neutral and MHC-linked microsatellite markers in 4 marginal populations of Atlantic salmon.

River/decade	Microsatellite Locus			Ssa171	Ssa197	Ssa202	SsaA124	SsaD144b	SsaD486	Ssasl438	Ssosl85	Sssp1605	Sssp2210	Avg	MHC-linked markers	
	CTAX	Est47	HSF												Sasa-DAA	Sasa-UBA
Asón 1950s																
N	25	24	25	22	20	21	25	25	25	25	24	23	24		45	45
AR	5.20	3.47	3.68	5.70	5.74	2.98	2.92	6.55	1.00	2.79	3.62	4.08	3.73	3.96	4.11	6.06
Ho	0.720	0.875	0.600	0.773	0.750	0.571	0.520	0.880	0.000	0.560	0.667	0.565	0.625	0.62	0.64	0.80
He	0.740	0.615	0.663	0.814	0.805	0.652	0.554	0.853	0.000	0.493	0.649	0.608	0.565	0.62	0.67	0.75
HW	0.000	0.061	0.430	0.823	0.048	0.108	0.951	0.951		0.975	0.979	0.036	0.901		0.157	0.059
Asón 1960s																
N	18	18	14	15	13	13	17	18	18	18	18	16	18		51	51
AR	4.12	3.89	3.97	4.12	4.58	3.18	2.35	6.82	1.33	3.05	4.09	4.30	4.36	3.86	2.825	6.7
Ho	0.444	0.667	0.857	0.533	0.538	0.462	0.588	0.889	0.056	0.722	0.611	0.500	0.722	0.58	0.58	0.85
He	0.657	0.622	0.648	0.664	0.728	0.601	0.517	0.858	0.054	0.583	0.702	0.691	0.640	0.61	0.5	0.63
HW	0.000	0.999	0.170	0.688	0.118	0.803	0.598	0.904	0.033	0.658	0.354	0.481	0.002		0.797	0.003
Asón 1980s																
N	32	32	32	32	32	28	32	32	32	32	31	32	32		32	32
AR	4.97	3.23	5.59	6.06	6.73	4.01	2.95	7.82	1.19	2.53	5.91	2.53	5.28	4.52	5.49	6.23
Ho	0.844	0.688	0.781	0.656	0.813	0.714	0.438	0.906	0.031	0.500	0.742	0.531	0.656	0.64	0.78	0.79
He	0.737	0.610	0.783	0.813	0.857	0.675	0.577	0.896	0.031	0.543	0.823	0.509	0.756	0.66	0.56	0.84
HW	0.977	0.953	0.963	0.694	0.692	0.003	0.928	0.338	0.924	0.995	0.001	0.001	0.000		0.009	0.886
Asón 1990s																
N	55	55	54	53	54	38	55	55	55	55	49	55	55		74	74
AR	5.01	3.36	6.08	6.23	6.78	4.82	3.20	7.91	1.51	4.06	5.58	3.54	4.50	4.81	5.4	6.97
Ho	0.582	0.727	0.741	0.736	0.815	0.605	0.527	0.782	0.073	0.673	0.776	0.582	0.782	0.65	0.76	0.85
He	0.728	0.611	0.814	0.837	0.856	0.752	0.581	0.899	0.103	0.691	0.810	0.621	0.737	0.70	0.56	0.79
HW	0.000	0.044	0.009	0.113	0.017	0.996	0.080	0.029	0.128	0.264	0.346	0.937	0.000		0.001	0.421
Asón 2002																
N	21	21	21	21	21	21	21	21	21	21	21	20	21		34	34
AR	4.55	4.16	6.01	7.11	7.38	4.64	3.39	7.68	1.78	3.84	5.16	3.38	5.72	4.99	5.99	6.25
Ho	0.667	0.714	0.905	0.857	0.905	0.714	0.571	0.905	0.143	0.762	0.857	0.700	0.952	0.74	0.81	0.81
He	0.649	0.702	0.817	0.859	0.874	0.751	0.590	0.889	0.135	0.629	0.778	0.628	0.825	0.70	0.85	0.81
HW	0.574	0.940	0.180	0.883	0.838	0.054	0.740	0.659	0.989	0.660	0.755	0.321	0.715		0.056	0.191
Deva 1950s																
N	20	20	20	19	17	18	20	20	20	20	20	19	20		32	32
AR	5.51	2.93	6.05	5.08	6.02	2.89	2.81	7.63	1.30	2.77	5.40	3.17	5.12	4.36	3.45	6.77
Ho	0.650	0.550	0.650	0.579	0.647	0.500	0.550	0.900	0.050	0.650	0.850	0.474	0.850	0.61	0.62	0.84
He	0.784	0.591	0.806	0.734	0.822	0.529	0.531	0.883	0.049	0.554	0.761	0.590	0.780	0.65	0.41	0.76
HW	0.468	0.837	0.648	0.599	0.018	0.735	0.768	0.464	0.909	0.021	0.056	0.942	0.514		0.001	0.079
Deva 1960s																
N	12	13	9	7	6	7	12	13	13	13	8	9	12		31	31
AR	4.74	3.39	7.79	3.70	6.00	3.99	4.02	8.48	1.00	2.92	5.94	2.90	4.71	4.58	5.86	5.85
Ho	0.333	0.615	0.222	0.286	0.333	0.286	0.333	0.846	0.000	0.385	0.375	0.000	0.750	0.37	0.85	0.78
He	0.649	0.612	0.877	0.459	0.694	0.724	0.479	0.896	0.000	0.544	0.742	0.494	0.726	0.61	0.17	0.33
HW	0.000	0.505	0.018	0.296	0.012	0.082	0.071	0.734		0.710	0.046	0.644	0.000		0.000	0.000
Deva 1980s																
N	44	44	40	44	44	41	42	44	44	44	42	29	44		44	44
AR	4.33	3.05	6.14	5.96	6.51	3.88	2.82	7.71	1.14	2.88	5.72	2.79	5.21	4.47	4.18	6.11
Ho	0.636	0.636	0.675	0.682	0.818	0.439	0.500	0.864	0.023	0.523	0.810	0.345	0.795	0.60	0.66	0.80
He	0.706	0.591	0.844	0.771	0.858	0.657	0.450	0.892	0.022	0.551	0.805	0.555	0.781	0.65	0.54	0.63
HW	0.000	0.114	0.047	0.483	0.949	0.010	0.939	0.002	0.439	0.000	0.984	0.000	0.000		0.002	0.001
Deva 1990s																
N	40	40	37	40	40	40	40	40	40	40	40	34	40		40	40
AR	4.86	3.14	6.62	6.51	6.49	4.42	3.54	8.45	1.00	3.12	5.37	3.07	4.66	4.71	4.88	6.03
Ho	0.725	0.525	0.865	0.875	0.850	0.650	0.475	0.925	0.000	0.675	0.750	0.559	0.725	0.66	0.72	0.82
He	0.681	0.601	0.864	0.818	0.853	0.704	0.536	0.913	0.000	0.601	0.774	0.575	0.759	0.67	0.43	0.8
HW	0.000	1.000	0.001	0.630	0.409	0.512	0.001	0.271		0.016	0.019	0.777	0.707		0.000	0.977
Deva 2002																
N	29	29	29	29	27	29	29	29	28	29	28	29	29		32	32
AR	5.72	3.19	6.56	6.26	6.55	4.54	2.98	7.36	1.21	3.18	5.57	3.85	3.89	4.68	4.85	6.95
Ho	0.759	0.621	0.897	0.897	0.926	0.517	0.379	0.862	0.036	0.690	0.821	0.724	0.828	0.69	0.77	0.85
He	0.783	0.589	0.861	0.809	0.853	0.698	0.382	0.875	0.035	0.596	0.803	0.570	0.710	0.66	0.88	1
HW	0.821	0.998	0.848	0.101	0.390	0.163	0.253	0.020	0.923	0.341	0.955	0.727	0.897		0.770	0.264
Nansa 1960s																
N	27	29	22	26	15	20	28	29	29	29	29	26	27		47	47
A	8	3	9	6	10	6	4	12	1	2	10	4	6		5.78	7.18
Ho	0.556	0.655	0.773	0.500	0.800	0.500	0.464	0.828	0.000	0.069	0.586	0.346	0.519	0.51	0.72	0.84
He	0.789	0.527	0.822	0.623	0.856	0.733	0.416	0.805	0.000	0.067	0.749	0.425	0.536	0.57	0.52	0.58
HW	0.000	0.391	0.298	0.129	0.316	0.013	0.971	0.727		0.847	0.003	0.312	0.982		0.013	0.000
Nansa 1980s																
N	29	29	29	28	28	25	29	29	29	29	26	29	29		31	31
AR	4.43	2.00	6.14	5.46	7.21	4.20	2.40	7.86	1.41	3.73	5.00	4.26	4.07	4.47	3.29	6.62
Ho	0.724	0.414	0.862	0.714	0.786	0.440	0.310	0.793	0.069	0.655	0.731	0.655	0.690	0.60	0.53	0.77
He	0.634	0.499	0.848	0.750	0.883	0.679	0.299	0.898	0.067	0.551	0.734	0.730	0.624	0.63	0.54	0.83
HW	0.982	0.356	0.301	0.988	0.236	0.008	0.769	0.264	0.998	0.992	0.957	0.508	0.912		0.565	0.165
Nansa 1990s																
N	21	21	12	21	21	20	13	21	21	21	20	21	21		32	32
AR	5.80	2.65	5.93	6.91	7.74	3.87	2.40	7.59	1.29	3.18	5.83	2.78	5.07	4.69	3.66	6.53
Ho	0.619	0.619	0.667	0.952	0.857	0.550	0.385	0.905	0.048	0.381	0.800	0.524	0.714	0.62	0.55	0.80
He	0.794	0.557	0.809	0.839	0.888	0.649	0.322	0.885	0.046	0.401	0.779	0.518	0.746	0.63	0.57	0.67
HW	0.152	0.907	0.145	0.551	0.617	0.996	0.864	0.223	0.911	0.745	0.735	0.988	0.302		0.734	0.006
Nansa 2002																
N	31	31	30	31	31	31	31	31	31	31	31	30	31		32	32
AR	4.82	3.07	5.85	6.25	7.02	5.11	3.28	8.49	1.48	2.90	6.55	3.57	5.04	4.88	5.18	6.57
Ho	0.710	0.548	0.833	0.935	0.806	0.774	0.677	0.903	0.097	0.452	0.903	0.767	0.871	0.71	0.75	0.74
He	0.720	0.594	0.826	0.824	0.875	0.784	0.586	0.915	0.092	0.421	0.830	0.640	0.771	0.68	0.69	0.78
HW	0.000	0.252	0.404	0.827	0.999	0.065	0.862	0.850	0.777	0.148	0.954	0.754	0.460		0.559	0.852
Pas 1950s																
N	29	29	27	25	23	21	26	28	28	29	26	24	29		67	67
AR	4.31	1.99	5.37	5.36	4.34	3.48	2.86	6.45	1.00	1.70	4.38	2.84	3.44	3.65	3.26	5.71
Ho	0.483	0.414	0.667	0.680	0.609	0.619	0.423	0.786	0.000	0.103	0.654	0.292	0.690	0.49	0.57	0.70
He	0.655	0.400	0.774	0.754	0.633	0.652	0.576	0.855	0.000	0.158	0.717	0.601	0.646	0.57	0.39	0.45
HW	0.000	0.006	0.847	0.052	0.917	0.127	0.364	0.355		0.064	0.334	0.815	0.002		0.013	0.000
Pas 1960s																
N	27	28	25	26	18	21	25	28	27	27	28	28	27		74	74
AR	4.42	2.09	5.40	4.92	5.85	4.66	2.07	6.29	1.00	1.54	4.82	2.63	4.89	3.89	3.93	5.51
Ho	0.481	0.286	0.800	0.538	0.667	0.619	0.280	1.000	0.000	0.111	0.786	0.500	0.889	0.54	0.64	0.70
He	0.615	0.275	0.796	0.692	0.736	0.740	0.246	0.844	0.000	0.105	0.765	0.554	0.776	0.55	0.54	0.44
HW	0.000	0.774	0.087	0.016	0.345	0.026	0.882	0.007		0.760	0.253	0.859	0.436		0.021	0.000
Pas 1980s																
N	62	63	63	56	62	60	63	62	63	63	26	62	63		63	63
AR	5.47	2.79	5.81	5.13	5.77	4.74	2.53	7.87	1.18	2.96	4.13	2.28	4.28	4.23	4.38	5.02
Ho	0.871	0.413	0.841	0.786	0.790	0.700	0.381	0.823	0.032	0.508	0.500	0.484	0.651	0.60	0.66	0.61
He	0.793	0.428	0.835	0.734	0.829	0.759	0.376	0.907	0.031	0.463	0.661	0.469	0.664	0.61	0.6	0.63
HW	0.000	0.977	0.511	0.987	0.869	0.419	0.998	0.554	0.898	0.574	0.784	0.156	0.000		0.044	0.413
Pas 1990s																
N	39	39	38	39	39	38	38	39	39	39	16	39	39		39	39
AR	6.30	3.66	6.25	5.31	6.22	5.46	3.49	8.71	1.50	3.69	4.47	4.00	5.11	4.93	5.77	6.15
Ho	0.769	0.487	0.921	0.667	0.795	0.763	0.500	0.923	0.051	0.692	0.438	0.667	0.821	0.65	0.8	0.77
He	0.825	0.615	0.831	0.752	0.847	0.792	0.606	0.924	0.097	0.671	0.633	0.637	0.780	0.69	0.84	0.62
HW	0.000	0.000	0.001	0.536	0.009	0.981	0.968	0.304	0.003	0.096	0.343	0.191	0.918		0.164	0.033
Pas 2002																
N	31	31	28	31	29	31	31	31	31	31	31	30	31		34	34
AR	5.20	3.88	5.26	4.79	6.17	3.29	3.13	7.75	1.00	2.86	5.71	3.55	5.66	4.48	5.58	5.3
Ho	0.774	0.613	0.786	0.645	0.862	0.613	0.548	0.871	0.000	0.710	0.839	0.633	0.839	0.67	0.73	0.63
He	0.780	0.679	0.781	0.697	0.831	0.618	0.585	0.897	0.000	0.533	0.796	0.659	0.825	0.67	0.73	0.68
HW	0.000	0.448	0.029	0.654	0.745	0.102	0.670	0.960		0.371	0.161	0.892	0.993		0.591	0.714

N = sample size, AR = allelic richness, Ho = observed heterozygosity, He = expected heterozygosity, HW = probability of conforming Hardy-Weinberg equilibrium, values in bold are significant after Bonferroni correction for multiple tests.

**Table 2:** Temporal and spatial AMOVA of population structuring in 4 marginal populations of Atlantic salmon (rivers Ason, Nansa, Pas and Deva in Northern Spain) based on 13 microsatellite markers.

Temporal structuring	Source of variation	% variance	F_ST_	*p*
Temporal stability, Asón	Among samples	0.87	0.009	<0.001
	Within river	99.13		
Temporal stability, Deva	Among samples	−0.48	−0.005	0.993
	Within river	100.48		
Temporal stability, Nansa	Among samples	0.02	0.000	0.478
	Within river	99.98		
Temporal stability, Pas	Among samples	2.05	0.020	<0.001
	Within river	97.95		
**Spatial structuring**				
Among rivers 1950s	Among populations	4.93	0.049	<0.001
	Within populations	95.07		
Among rivers 1960s	Among populations	5.72	0.057	<0.001
	Within populations	94.28		
Among rivers 1980s	Among populations	1.61	0.016	<0.001
	Within populations	98.39		
Among rivers 1990s	Among populations	0.22	0.002	0.167
	Within populations	99.78		
Among rivers 2002	Among populations	0.49	0.005	0.047
	Within populations	99.51		

**Table 3:** Temporal and spatial genetic structuring based on F_ST_ in 4 marginal populations of Atlantic salmon (rivers Ason, Nansa, Pas and Deva in Northern Spain), estimated using MHC-linked markers.

Temporal samples	Sasa-DAA-3′UTR	Sasa-UBA-3′UTR
Asón	0.0143***	0.0121**
Deva	0.0179**	0.0204*
Nansa	0.0088	0.0073
Pas	0.0357***	0.0458***
**Spatial samples**		
1950s	0.023	0.070***
1960s	0.034*	0.055***
1980s	0.028***	0.044***
1990s	0.030***	0.020***
2002	0.020***	0.053***

*p < 0.05 **p < 0.01 ***p < 0.001.
