# Supplementary material for: Temporal and spatial instability in neutral and adaptive (MHC) genetic variation in marginal salmon populations
Source: Sci Rep. 2017 Feb 10;7:42416. doi: 10.1038/srep42416 (PMC5301200; doi:10.1038/srep42416)
Supplement: Supplementary Information [file srep42416-s1.pdf]

Supplementary material

Temporal and spatial instability in neutral and adaptive (MHC) genetic variation of marginal salmon populations

Kate L. Ciborowski[1], William C. Jordan[1], Carlos García de Leániz[2], Sofía Consuegra[2]

Figure S1. STRUCTURE clustering of Atlantic salmon adult samples from four marginal populations in Northern Spain over four decades analysed per river of origin. Each individual is represented by a vertical bar of a colour that represents its estimated membership to one of 6 genetic clusters (q). Labels below the plot indicate decade and K is the most likely number of genetic groups.

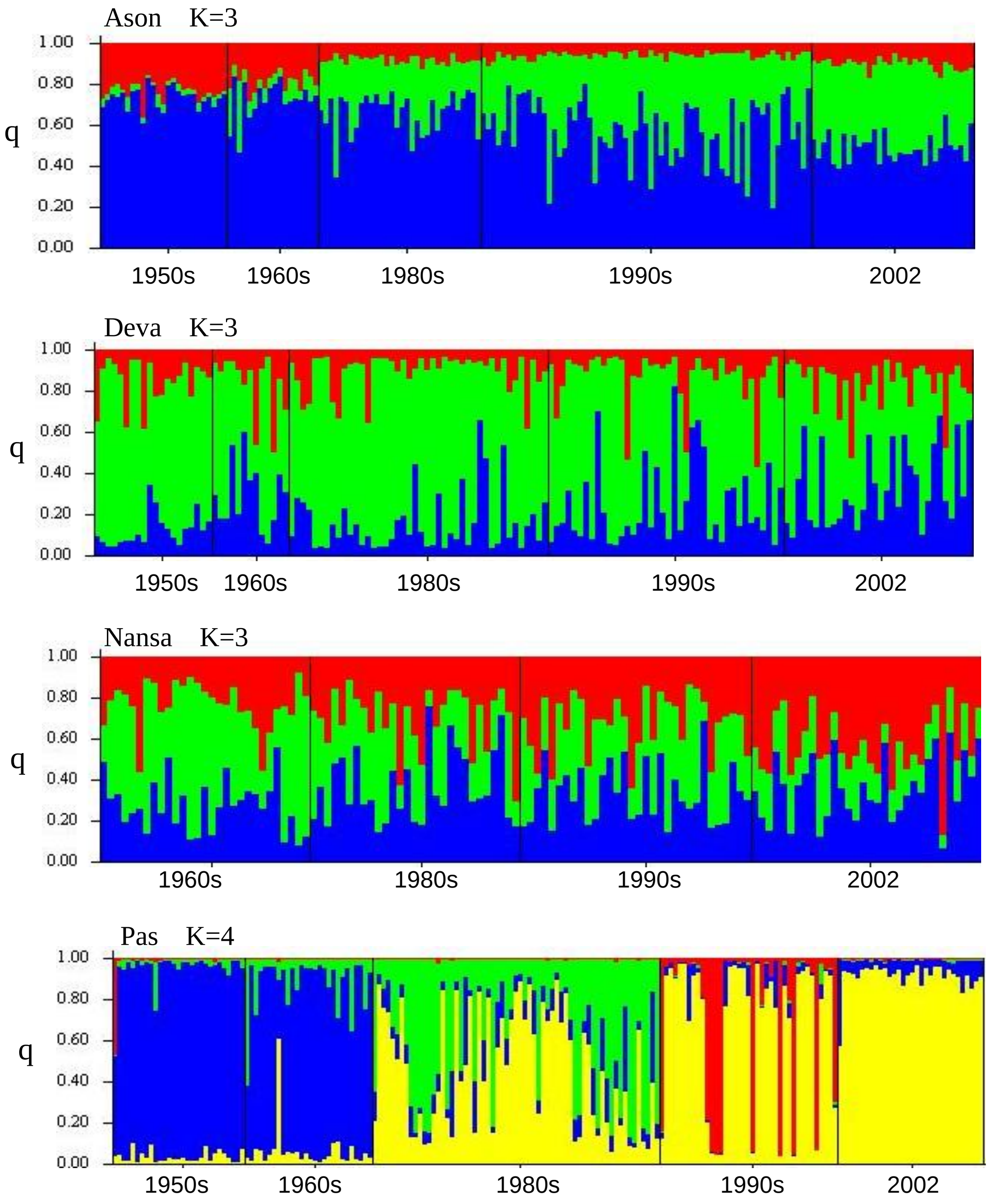

Figure S2. Allele frequency of MHC-linked markers in four 4 marginal populations of Atlantic salmon (rivers Ason, Nansa, Pas and Deva in Northern Spain): (a) *Sasa-UBA-3'UTR*; (b) *Sasa-DAA-3'UTR*.

(a)

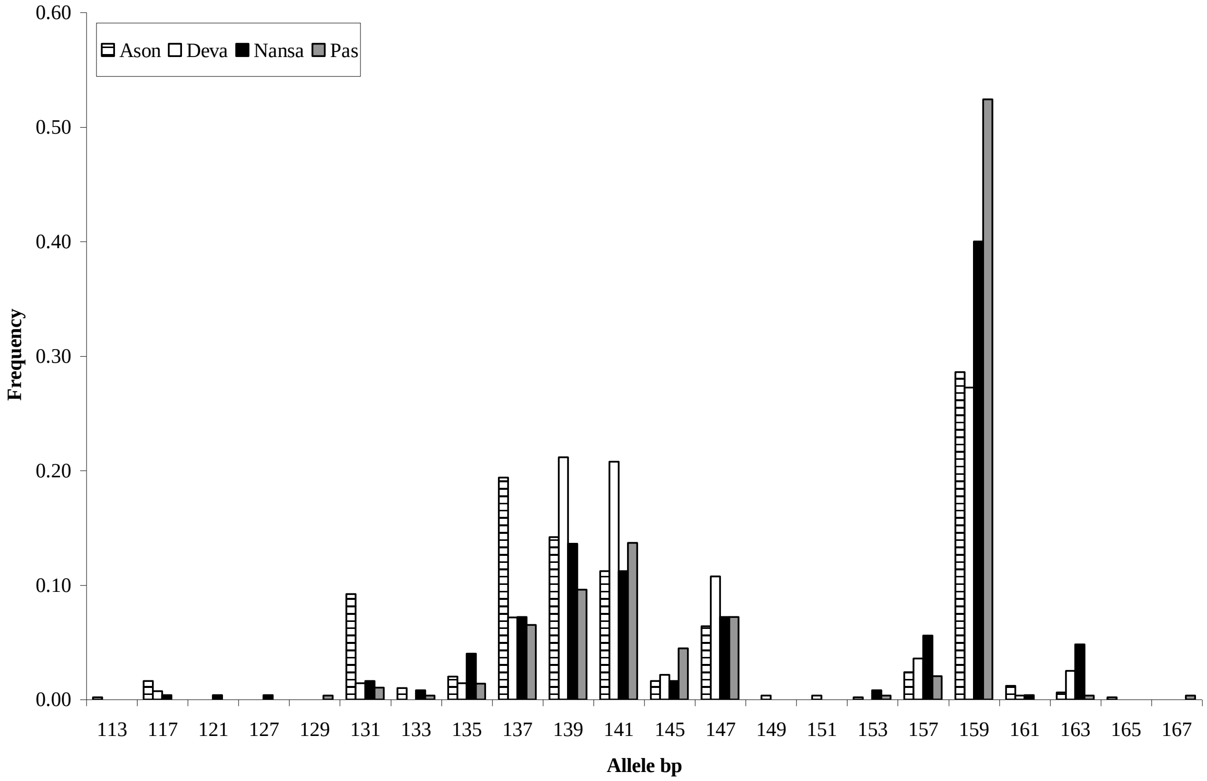

(b)

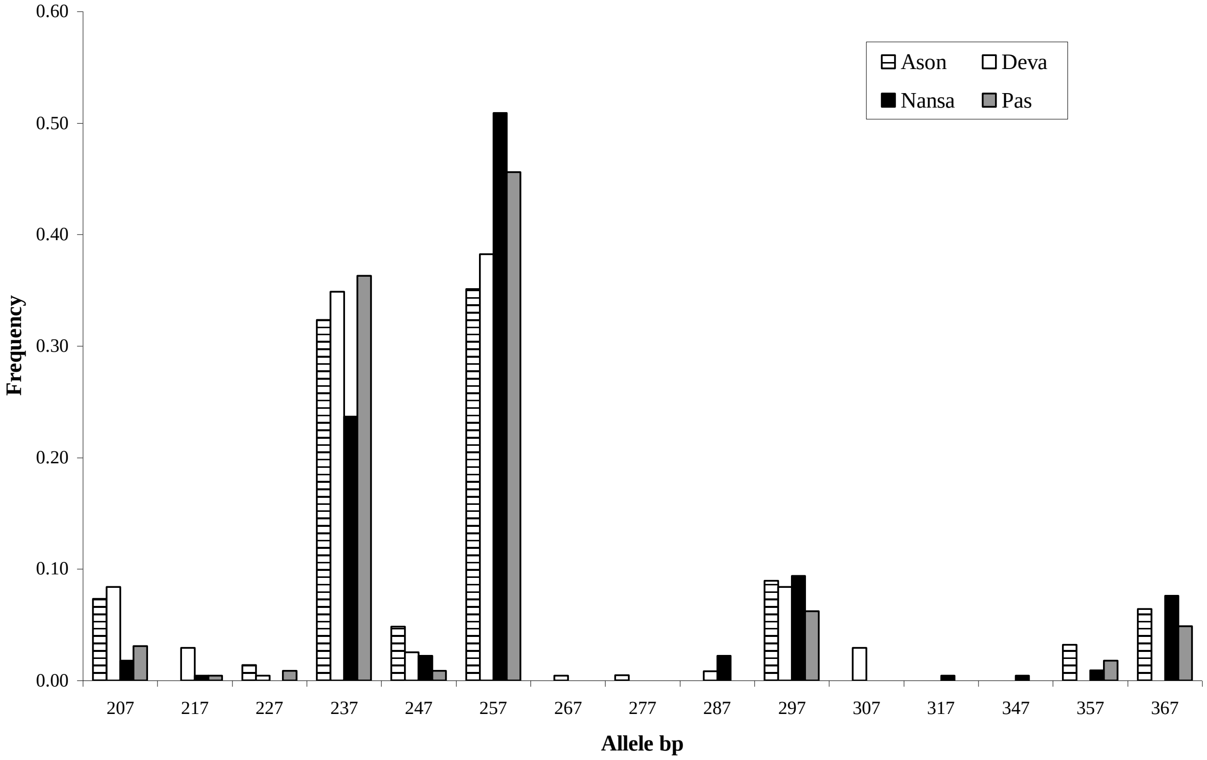
Frequency
0.60
0.50
0.40
0.30
0.20
0.10
0.00
Ason
Deva
Nansa
Pas
207
217
227
237
247
257
267
277
287
297
307
317
347
357
367
Allele bp

Table S1. Pearson correlation coefficient in allele frequencies between pairs of temporal samples for each of the population for neutral microsatellites and MHC-linked markers. ** P<0.01; *** P<0.001, in black and italics are those significant after strict Bonferroni correction for 45 tests.

(a)

Comparison	*Microsatellites*		*Sasa-UBA-3'UTR*		*Sasa-DAA-3'UTR*	
	r	df	*r*	df	*r*	df
A50vA60	0.93**	105	0.88***	11	0.89**	3
A60vA80	0.91**	120	0.85***	12	0.97***	5
A80vA90	0.95**	144	0.93***	12	0.92***	7
A90vA02	0.94**	147	0.80***	10	0.85***	8
D50vD60	0.84**	120	0.82***	9	0.40	4
D60vD80	0.88**	134	0.61**	11	0.55	6
D80vD90	0.96**	137	0.93***	11	0.99***	7
D90vD02	0.95**	137	0.91***	10	0.86***	7
N60vN80	0.91**	128	0.78***	12	0.98***	9
N80vN90	0.95**	125	0.92***	9	0.99***	5
N90vN02	0.94**	135	0.90***	11	0.91***	6
P50vP60	0.97**	103	0.98***	11	0.98***	7
P60vP80	0.92**	115	0.98***	10	0.91***	6
P80vP90	0.91**	137	0.94***	10	0.87***	7
P90vP02	0.89**	132	0.91***	9	0.82***	8
